# Novel approach for tracking interdisciplinary research productivity using institutional databases

**DOI:** 10.1017/cts.2022.455

**Published:** 2022-08-30

**Authors:** E. Bengert, L. Towle-Miller, J. Boccardo, G. Mercene, P.J. Ohtake, P. Balkundi, P.L. Elkin, J. Balthasar, T.F. Murphy, K. Noyes

**Affiliations:** 1 University at Buffalo Clinical and Translational Science Institute, Buffalo, NY, USA; 2 GlaxoSmithKlein, Philadelphia, PA, USA; 3 Department of Biostatistics, School of Public Health and Health Professions, University at Buffalo, Buffalo, NY, USA; 4 CB Insights, Buffalo, NY, USA; 5 Department of Rehabilitation Science, School of Public Health and Health Professions, University at Buffalo, Buffalo, NY, USA; 6 Organization and Human Resources Department, School of Management, University at Buffalo, Buffalo, NY, USA; 7 Department of Biomedical Informatics, Jacobs School of Medicine and Biomedical Sciences, University at Buffalo, Buffalo, NY, USA; 8 Department of Pharmaceutical Sciences, School of Pharmacy and Pharmaceutical Sciences, University at Buffalo, Buffalo, NY, USA; 9 Department of Medicine, Jacobs School of Medicine and Biomedical Sciences, University at Buffalo, Buffalo, NY, USA; 10 Department of Epidemiology and Environmental Health, School of Public Health and Health Professions, University at Buffalo, Buffalo, NY, USA

**Keywords:** Interdisciplinary team science, scientific publications, research grants, program evaluation, temporal trends

## Abstract

This study proposes a new practical approach for tracking institutional changes in research teamwork and productivity using commonly available institutional electronic databases such as eCV and grant management systems. We tested several definitions of interdisciplinary collaborations based on number of collaborations and their fields of discipline. We demonstrated that the extent of interdisciplinary collaboration varies significantly by academic unit, faculty appointment and seniority. Interdisciplinary grants constitute 24% of all grants but the trend has significantly increased over the last five years. Departments with more interdisciplinary grants receive more research funding. More research is needed to improve efficiency of interdisciplinary collaborations.

## Introduction

Over the past two decades, interest and investments in large-scale interdisciplinary team projects have grown dramatically, evident by the steep growth in interdisciplinary publications, journals, requests for proposals by public agencies and foundations, and research collaborations [1,2]. The increasing complexity of scientific problems and emphasis on research translation to practice are the main motivations behind interdisciplinary research [3–6]. Collaborative and interdisciplinary Team Science Cores have been essential in the functioning of all CTSA hubs [7]. Multi- and interdisciplinary Team Science Cores are woven through all CTSA cores to foster an environment that promotes scientific innovation through interdisciplinary alliances, equipping investigators with skills and resources to communicate with partners within and outside of their own area of expertise (including academic, industry, and community partners). Team Science Cores also develop and implement numerous innovative strategies to train researchers how to be effective leaders and contributors in research teams including approaches aimed at increasing diversity and inclusion in clinical and translational research. However, evaluation of the impact of these activities – whether they have moved the needle in the right direction and facilitated formation and functioning of new effective interdisciplinary research teams – have received limited attention [8].

We define teamwork as the set of behaviors by two or more interdependent individuals as a function of coordinating requirements imposed by independent tasks in achieving common goals [9]. Better, more efficient teamwork is expected to lead to higher team productivity, greater innovation, and faster dissemination of research findings into community practice. Team performance is a multidimensional construct, indicating that evaluation of teamwork requires capturing several distinct metrics using a variety of tools and approaches, such as surveys of team members and nonmembers, analysis of publication authorship and grant submission systems, human resource databases, department activity schedules, and others [2,10–21].

While several validated measures of team performance are based on direct observation of team activities, these measures are not practical (take a long time to collect data and require large staff and budget) for the assessment of large numbers of teams (e.g., university-wide), especially research teams in post-COVID times that function asynchronously across several settings [8]. Evaluation strategies based on direct observation are also known to introduce observer bias and are more likely to identify outliers.

This study proposes a pragmatic approach for tracking institutional changes in research teamwork and productivity in real time using common institutional electronic databases such as eCV and grant management systems. Broad national dissemination of this approach across other CTSA hubs could provide a standard metric for comparing teamwork productivity across different programs and academic units, facilitate our ability to assess effectiveness of various team-training activities, and improve quality and value in interdisciplinary research. Implementation of best practices in interdisciplinary team science and strong collaborations with communities, special populations, patients, and stakeholders will enable high-impact, innovative research to improve the health of our communities and nation.

## Methods

### Sources of Data

Data on faculty publications were obtained from the institutional eCV online Faculty Profile system from 2018 to 2021. Faculty profiles were created to allow faculty and clinical providers to specify their Curriculum Vitae (CV) information in one electronic location so that the information could be collected for many purposes including NIH Biosketches and institutional annual reports. Data on faculty grants were obtained from the institutional grant management system CLICK from 2015 to 2020. The CLICK Portal software integrates all aspects of grants management into a single system, including Institutional Review Board approvals, animal studies, grant management, financial conflict of interest, research agreements, and safety. The database included information on the proposal number, grant PI, co-Is, credited departments and percent effort, submission status (new, resubmission, renewal), proposal state (funded or not), project sponsors, submission date and title, as well as the award amount.

### Faculty Publication Data

Using the institutional eCV database (n = 1300) available at three health sciences schools, we selected a purposeful (diverse) sample of clinical, basic science, and translational science departments that included departments of Biochemistry and Medicine (School of Medicine), Pharmaceutical Sciences (School of Pharmacy), and Oral Biology and Community and Pediatric Dentistry (School of Dentistry) (n = 340). In the department of Medicine, faculty were further divided into divisions. We excluded individuals who did not have a faculty appointment (postdocs or instructors) and who had less than 5 publications in the last 3 years to allow for an accurate assessment of faculty teamwork. These cutoffs were set to optimize faculty inclusion/exclusion criteria, institutional generalizability, and statistical stability. The research team agreed that a researcher’s pattern of collaboration could be defined with reasonable validity based on their random set of 5 publications. While more data (more publications over longer period of time) are more desirable for statistical power, excluding faculty with fewer publications over shorter period of time would bias the sample against junior and newly hired faculty. A similar approach is used for clinical quality assessment [22].

By the type of appointment, the faculty were categorized as tenure track vs nontenure track/clinical. After excluding nonfaculty appointments, faculty with less than five publications on Web of Science, and individuals with missing information on appointment status, the final analytic sample included n = 187 faculty.

### Evaluating Interdisciplinary Publications

To quantify each faculty propensity for interdisciplinary research, we collected data from the five most recent peer-reviewed publications per faculty member identified from the Web of Science Core Collection. We recorded the publication’s number of authors, number of unique organizations they represent, and the number of unique fields of expertise (e.g., biochemistry, gene sequencing) based on each author’s affiliation. We also recorded the total number of publications per faculty from 2018 to 2021. Publications were excluded if organizations and/or fields were not listed in the database, if the publication only had one author, and if a paper had a large number of authors (>20). Publication characteristics were compared by faculty seniority and appointment type using nonparametric Wilcoxon Rank Sum Test (p < 0.05).

### Grant Data

Our grant analysis was based on the proposal data from the years 2015–2020 (n = 10,106) from all decanal units at the institution. Interdisciplinary proposals were defined as proposals with multiple investigators from multiple departments. Comparisons were made between interdisciplinary versus single-discipline proposals in regard to the number of investigators, number of departments, type of sponsor, and amount awarded. The percentage of interdisciplinary proposals was summarized at the investigator and department level, and trends over time were explored.

## Results

### Publications Analysis

**Fig. 1.**
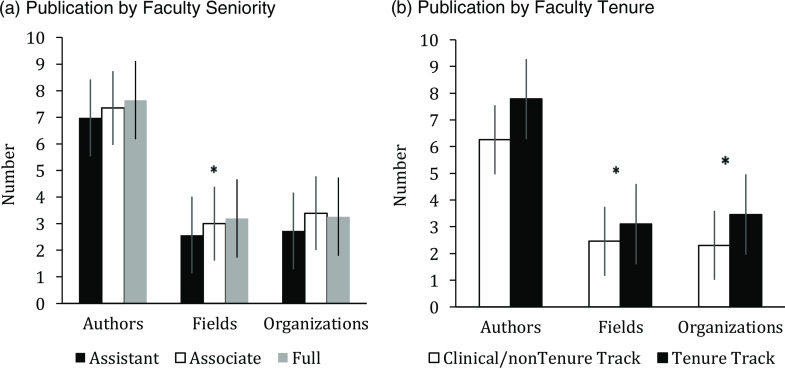
Faculty Appointment.

The sample of faculty (n = 205) was closely split among assistant (n = 79 or 39%), associate (61 or 30%) and full professors (65 or 32%), with two-thirds (68%) of the faculty being on tenure track. For this cohort, we identified 1,025 manuscripts published within the last 3 years (325 manuscripts by full professors, 305 by associate professors, and 395 by assistant professors). The higher the seniority of the faculty, the more interdisciplinary their publications tended to be (including 3.2 distinct fields of study for full professor, SD 1.3 vs 3.0 for associate, SD 1.2 vs 2.6 for assistant, SD 1.0, p < .01) (Fig. 1). Tenure track faculty collaborated with more organizations (3.5, SD 2.5 vs 2.3, SD 1.1, p < .01) and were more interdisciplinary (3.1, SD 1.2 vs 2.5, SD 1.0, p < .01) compared to clinical/nontenure track faculty. Faculty seniority and tenure track status had no significant effect on the number of co-authors per publication.

### Extent of Interdisciplinary Grant Collaborations Across Different Academic Units

Overall, there were 10,106 grant proposals submitted by faculty between 2015 and 2020. Three quarters of them (n = 7,646) were from a single discipline, with 87% of them involving only one investigator and 13% involving multiple investigators from the same department. There were 2,460 interdisciplinary proposals (24% of the total) with 2 or more investigators from different departments. The average number of investigators per proposal was 3.0 for interdisciplinary (SD 1.4) compared to 1.2 for monodisciplinary (SD 0.5).

**Fig. 2.**
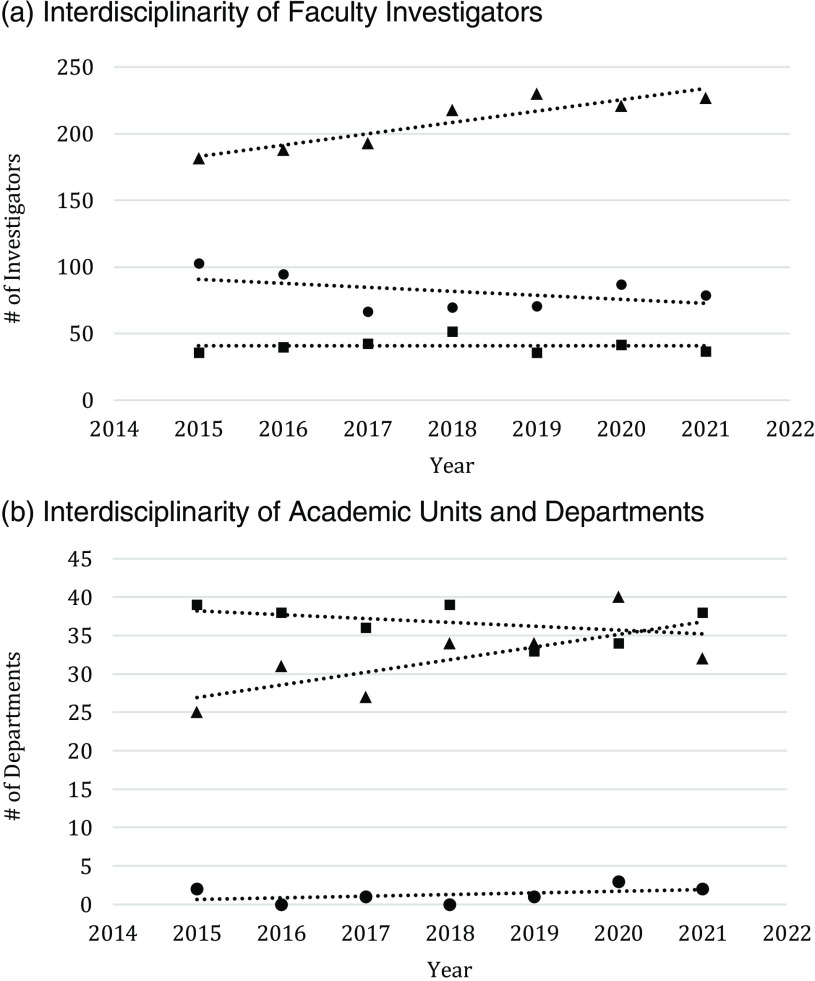
Trends in Interdisciplinary Productivity by Faculty (a) and Department (b). ● represent faculty/departments with no interdisciplinary proposals; ▪ represent faculty/departments with some interdisciplinary proposals; ▴ represent faculty/departments with all interdisciplinary proposals.

Fig. 2 displays the temporal trend in the interdisciplinarity of investigator and departmental research from 2015 to 2020 estimated using grant submissions. The proportion of investigators who submitted only interdisciplinary proposals (Fig. 2a) increased from 57% in 2015 to 66% in 2021. At the same time, the number of investigators submitting no interdisciplinary proposals decreased from 103 in 2015 (32%) to 79 in 2021 (23%). A similar trend was observed among departments with the number of departments submitting all interdisciplinary proposals raising from 25 (38%) in 2015 to 32 (44%) in 2021 (Fig. 2b). Funding source was significantly associated with the interdisciplinary nature of the studies with foundation and federal grants being significantly more interdisciplinary than grants funded by professional societies (70% vs 58%).

### Funding Levels and Interdisciplinarity

**Fig. 3.**
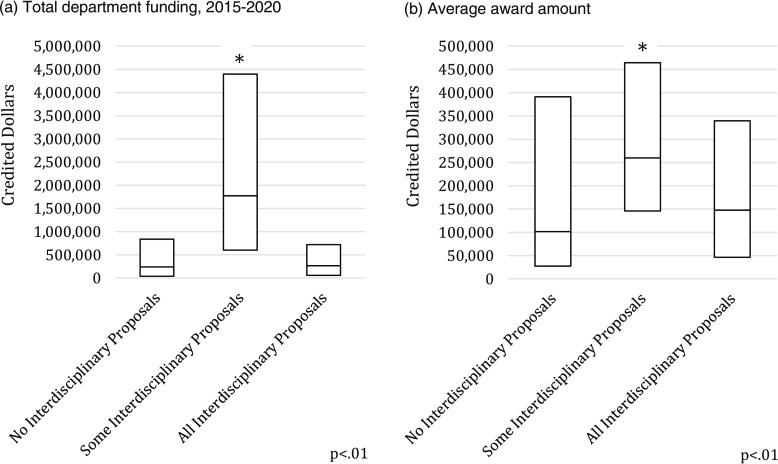
Variation in Department Funding, by interdisciplinarity status (excluding awards <$1,000).

Among all academic departments and decanal units that submitted at least one proposal in 2015–2020, 6% (n = 6) submitted only single discipline awards, 82% (n = 78) submitted both single and interdisciplinary awards, while the remaining 12% of the decanal units submitted only interdisciplinary proposals. Decanal units that submitted both single discipline and interdisciplinary proposals received the highest total funding (mean $1M, SD $2.1M) and had the highest amount awarded per grant (mean $396K, SD $1.6M) compared to the departments that submitted no interdisciplinary proposals (Fig. 3a and 3b). Departments that submitted both single discipline and interdisciplinary proposals submitted 731 awards for $1,000+ over the six-year period compared to 900 awards from departments that submitted no interdisciplinary proposals.

## Discussion

Our analysis has identified a significant increase in the prevalence of interdisciplinary collaborations across both individual investigators and academic units since 2015. We identified a significant association between the sources of extramural funding and the interdisciplinary nature of submitted proposals, with a significantly higher proportion of the NIH and NSF proposals including investigators from various academic units compared to local and private funding. Furthermore, interdisciplinary grants on average had higher budgets than single discipline proposals. That resulted in academic units that pursued interdisciplinary grant funding having larger grant portfolios compared to academic units with predominantly single-investigator single discipline projects.

We also identified that senior tenure-track faculty are engaged in more interdisciplinary collaborations compared to junior and non-tenure track investigators. This analysis was driven by two competing hypotheses: (1) Younger investigators are more likely to be risk takers and try new approaches outside of the traditional norms including interdisciplinary collaborations and (2) senior faculty are free from the constraints of tenure expectations and have a wider circle of colleagues from various disciplines formed through various administrative and professional activities, hence, their interdisciplinary collaborations should increase with time [23–25]. This finding emphasizes the importance of senior faculty in promoting institutional culture of collaboration but could also signal that there are potential barriers for junior investigator participation in interdisciplinary collaborative teams and networks.

Despite the widespread agreement on the importance and promises of interdisciplinary research teams (IDRT), barriers for effective IDRT implementation remain significant. Prior studies across a wide range of academic disciplines have identified several common barriers to IDRT including (1) university organization that favors mono-disciplinary departmental structure; [26] (2) tenure and promotion system that favors individual productivity; [27] (3) additional challenges posed by distance between collaborators (e.g., time spent in transit, scheduling conflict) [28], (4) difference in cultural norms across disciplines (e.g., ranking of research journals, importance of extramural funding, teaching/clinical responsibilities) [29], and (5) lack of experience in teamwork among researchers and research administrators (effective communication, team role vs organizational hierarchy, resource sharing, contract negotiations, identifying common goals/shared mental model) [30]. Overall, it is more difficult, expensive, and time consuming to work in research teams vs. solo research [5]. At the same time, there is growing evidence that IDRTs are associated with significant advantages such as greater practical impact, visibility, innovation, and potential to secure funding [8]. This evidence suggests that interdisciplinary teamwork should not be viewed as a “one size fits all” magic solution to academic productivity but rather as a strategic approach that should be used thoughtfully for research questions and organizations that are more likely to benefit from IDRT.

Developing efficient and sustainable mechanisms for tracking interdisciplinary teamwork in academia is extremely important to be able to evaluate and disseminate numerous programs that aim to promote interdisciplinary collaborations in research [31,32]. While several metrics for assessing use of team skills have been developed for experimental settings, these tools often require extensive observational data collection and participation of expert panels that are too labor-intensive to apply at organizational and programmatic levels and do not generate results in real time [2,10–21]. As a result, such tools have limited potential for helping guide development and implementation of organizational policies and curricula to support development of interdisciplinary team science at institutional and departmental levels. In this study, we relied on the approaches and best practices that have been used at the national level to adapt them for use in a single institution analysis [10,18,33]

Our study is unique in several important ways. To our best knowledge, this is the first study demonstrating an empirical approach to tracking the impact of programmatic and educational activities focused on promoting academic interdisciplinary collaboration and teamwork using existing institutional databases. Our findings also demonstrated one of many benefits of interdisciplinary collaboration in research – ability to attract greater amounts of extramural funding from the most prestigious sources reflected in national rankings of programs and academic institutions (the NIH and NSF awards). Finally, our findings that senior tenure track faculty have greater interdisciplinary collaborative networks point to a potentially important relationship between research interdisciplinarity and academic tenure and promotion. This finding is consistent with prior observations of Way et al. 2017 that academic productivity over one’s career is not always linear and could be affected by a variety of personal, discipline-specific, and organizational factors [34].

Despite the strengths and innovation of the approach presented here, the quantitative findings should be interpreted with caution and only as an example of potential applications for this approach. First, the study is based on a sample of departments, publications, and grants from a single institution which may limit the study generalizability across units and organizations. Because our analysis of publications only covered a relatively short time period, we cannot demonstrate causality between the extent of faculty interdisciplinary collaborations and their ability to receive tenure/promotion. Because of the relatively small number of grants and manuscripts submitted by individual investigators and some departments annually, our statistical analysis was limited and could not include subgroup analysis (e.g., by funding status, sub-contracts vs primary awards, and others) [10].

In summary, this study provides empirical evidence of the benefits of interdisciplinary collaboration in research and identifies an important role that senior faculty may be playing in creating the culture of interdisciplinary teamwork. Our findings suggest several possible mechanisms how interdisciplinary collaborations could facilitate diversity, equity, and inclusion in clinical and translational research including the role of funding sources and cultural norms within decanal units. More research is needed to help minimize barriers for interdisciplinary collaborations for junior investigators and in academic fields that traditionally do not receive federal research funding and engage in mainly single discipline scholarly pursuits.
